# An osteoid osteoma coexisting with a ganglion cyst in the fifth metatarsal head: A rare case report

**DOI:** 10.1016/j.radcr.2026.06.096

**Published:** 2026-07-14

**Authors:** Morteza Gholipour, Fatemeh Mousavi Eshlaghi, Maryam Almasi, Arman Namazi, Fatemeh Abbasi

**Affiliations:** a Clinical Research Development Unit of Akhtar Hospital, Shahid Beheshti University of Medical Sciences, Tehran, Iran; b School of Medicine, Student Research Committee, Shahid Beheshti University of Medical Sciences, Tehran, Iran; c School of Medicine, Tehran University of Medical Sciences, Tehran, Iran; d Department of Orthopedics, Urmia University of Medical Sciences, Urmia, Iran; e Department of Medicine, To.C., Islamic Azad University, Tonekabon, Iran

**Keywords:** Osteoid osteoma, Ganglion cysts, Metatarsal bones, Curettage

## Abstract

Osteoid osteoma is a small, benign, but intensely painful bone tumor that usually arises in the diaphysis or metaphysis of long bones such as the femur, tibia, or humerus. Its occurrence in the metatarsal bones is exceedingly rare. Even more unusual is its coexistence with a ganglion cyst, a benign cystic lesion commonly seen around joints or tendon sheaths. The overlapping symptoms of these 2 entities can mask the typical presentation of osteoid osteoma and make diagnosis challenging. We describe a 70-year-old man who presented with nocturnal pain localized to the head of the fifth metatarsal, which was temporarily relieved by nonsteroidal anti-inflammatory drugs. Magnetic resonance imaging (MRI) unexpectedly revealed an osteoid osteoma associated with a ganglion cyst in the same region. The patient underwent curettage and cauterization, leading to complete resolution of pain within 2 weeks. This rare presentation, particularly given the patient’s age of 70 years, underscores the need to maintain a high index of suspicion for osteoid osteoma, even when it occurs in atypical sites and in the presence of concurrent benign lesions such as a ganglion cyst. Recognizing this unusual coexistence can help avoid misdiagnosis and ensure timely, effective treatment.

## Introduction

Foot pain, affecting 14%-42% of people at any given time, is one of the most common complaints encountered in orthopedic clinics [1]. The etiology can range from simple soft-tissue conditions to bone tumors. Pain due to bone tumors such as osteoid osteoma remains rare and diagnostically challenging. In a review of 981 foot and ankle bone lesions, 79.41% were benign, and osteoid osteoma accounted for 28.3% of all benign bone tumors [2].

Osteoid osteoma is a relatively common benign bone tumor that is typically less than 1.5 cm in diameter. This condition usually presents with discomfort, especially nocturnal pain [3]. Although osteoid osteoma mostly affects individuals in late childhood, it can occasionally present in older patients and in atypical locations, such as the foot [4]. These unusual presentations may contribute to misdiagnosis and delay in treatment.

Ganglion cysts are the most common benign soft tissue masses of the hand and wrist [5]. They typically manifest as painless, asymptomatic swellings of the wrist and foot. However, atypical presentations have been reported in the tarsal tunnel and intermetatarsal space, as well as with complications such as inflammation or abscess formation. Such atypical cysts may obscure the diagnosis of coexisting lesions like osteoid osteoma. High-resolution magnetic resonance imaging (MRI) and ultrasound are valuable diagnostic tools for ganglion cysts, especially when the cyst coexists with other bone or soft tissue abnormalities [6].

Here, we present a rare case of osteoid osteoma of the fifth metatarsal bone in a 70-year-old man with the unusual co-occurrence of a ganglion cyst, which was successfully excised.

## Case report

A 70-year-old male presented to the hospital with intermittent ache and swelling in his right foot. The patient claimed he had had the pain for several years and had difficulty standing and walking. The pain worsened during the night and was alleviated with nonsteroidal anti-inflammatory drugs (NSAIDs). There was no history of concomitant trauma. The pain was localized in the head of the fifth metatarsal. In physical examination, the distal diaphysis was tender and swollen without erythema and calor (Fig. 1). Range of motion (RoM) was intact, and there were no signs of neurovascular defects. Initially, standard dorsoplantar (AP) and lateral radiographs of the foot were obtained. In general, there was no sign of fracture or dislocation with normal overall alignment. In lateral view, a small cyst-like lesion was seen (Fig. 2). Further imaging of the fifth metatarsal showed subtle cortical thickening along the shaft and a small lytic lesion in the axial view of computed tomography (Fig. 3). On MRI, on a T1 weighted image, small, round, low signal lesion in the cortex (nidus) was seen (Fig. 4).

**Fig. 1:**
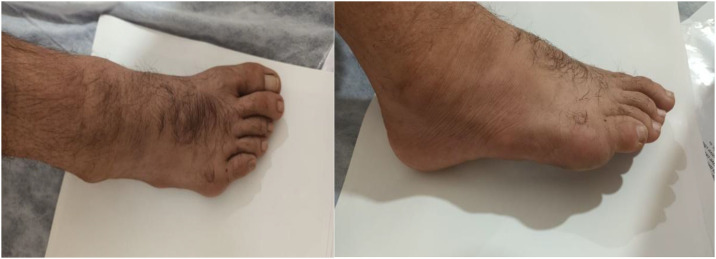
Clinical photograph of the right foot showing swelling localized over the head of the fifth metatarsal. No erythema, calor, or skin changes are evident.

**Fig. 2:**
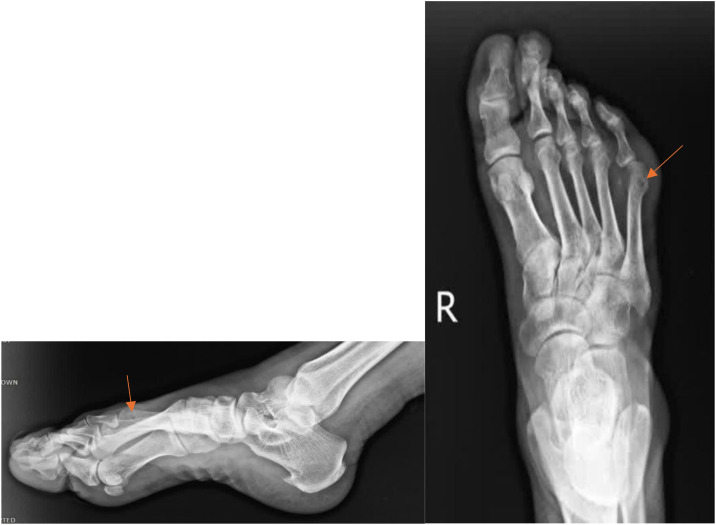
Standard dorsoplantar (AP) and lateral radiographs of the right foot. The overall alignment is preserved, with no fracture or dislocation. In the lateral view, a small cyst-like radiolucent lesion can be seen near the head of the fifth metatarsal.

**Fig. 3:**
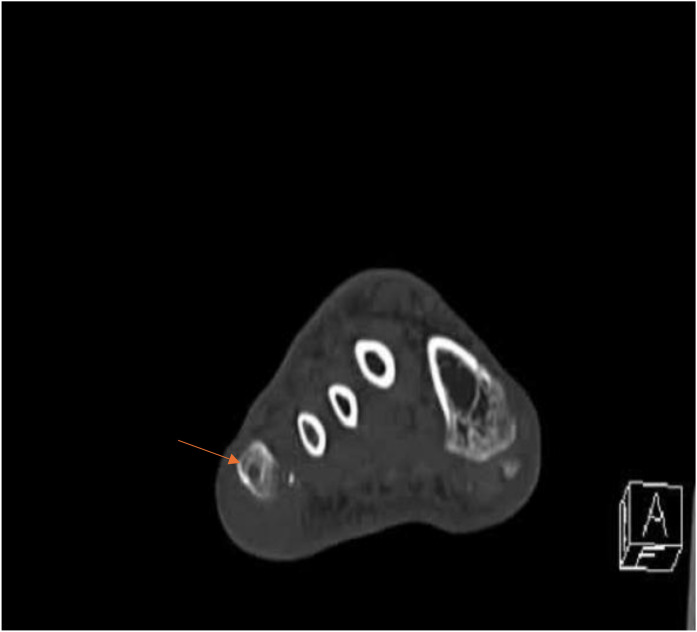
Axial computed tomography (CT) scan of the fifth metatarsal demonstrating subtle cortical thickening and a small lytic lesion with well-defined margins, consistent with a nidus of osteoid osteoma.

**Fig. 4:**
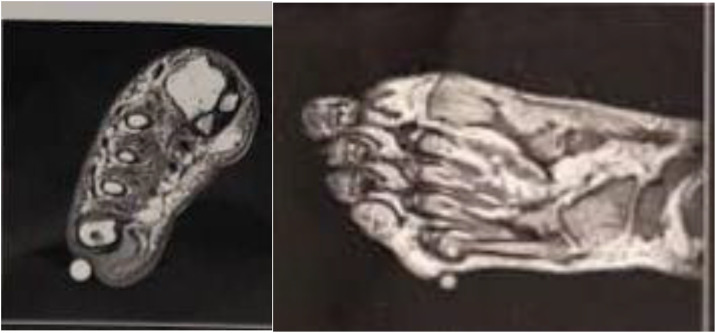
MRI of the right foot. T1-weighted axial image reveals a small, round, low-signal lesion within the cortex of the fifth metatarsal head (nidus).

In hematological assessments, inflammatory biomarkers were negative. Bone tumor biomarkers (Ca, Phos, LDH, and PTH) and PSA were also normal. There was no trace of bence jones protein in the urine.

The patient underwent excision of the soft tissue mass along with curettage of the cyst and cauterization of the inner walls (Fig. 5, Fig. 6).

**Fig. 5:**
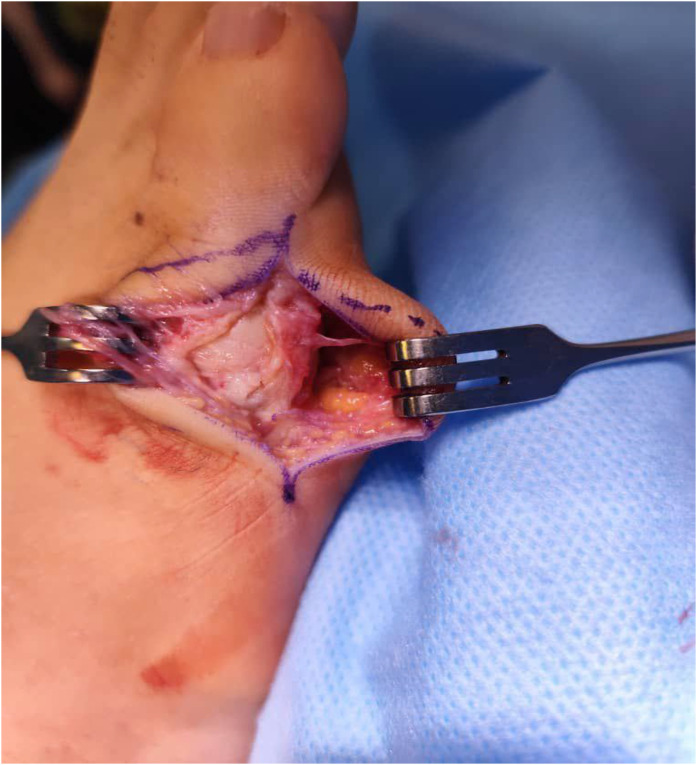
Intraoperative photograph showing curettage of the lesion at the head of the fifth metatarsal following dorsolateral incision.

**Fig. 6:**
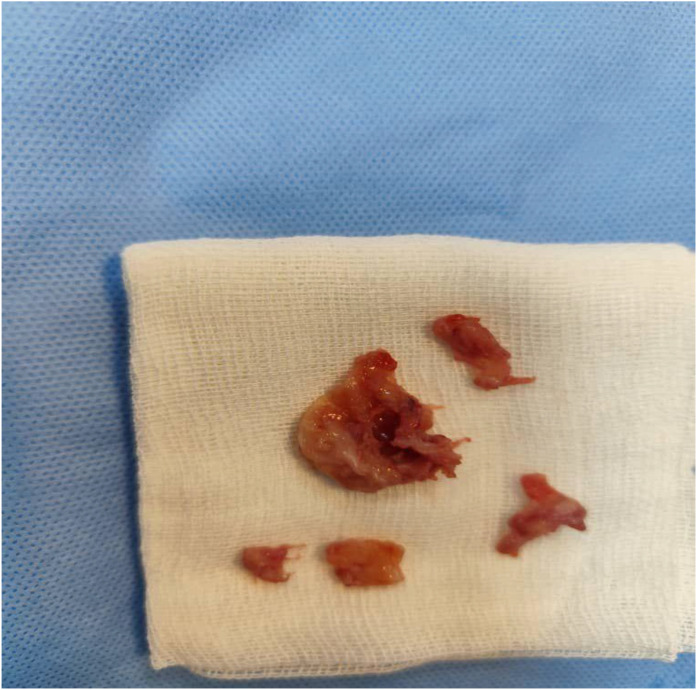
Excised intraosseous tumor tissue obtained during surgery.

A superficial dorsolateral incision was made over the head of the fifth metatarsal. The soft tissue cyst was excised along with its capsule (Fig. 7). At that point, the nidus and the site of the cyst were curetted. Then, the skin tissues were sutured. Pathological inspection of the excised mass revealed a ganglion cyst with an osteoid osteoma of the head of the fifth metatarsal (Fig. 8). Within 10 days after the operation, clinical symptoms resolved thoroughly (Fig. 9).

**Fig. 7:**
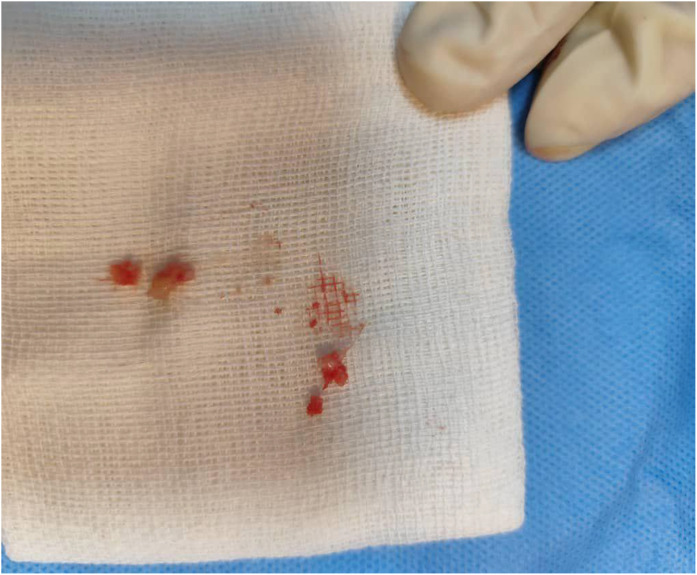
Gross specimen of the excised ganglion cyst along with its capsule, removed from the dorsolateral aspect of the fifth metatarsal.

**Fig. 8:**
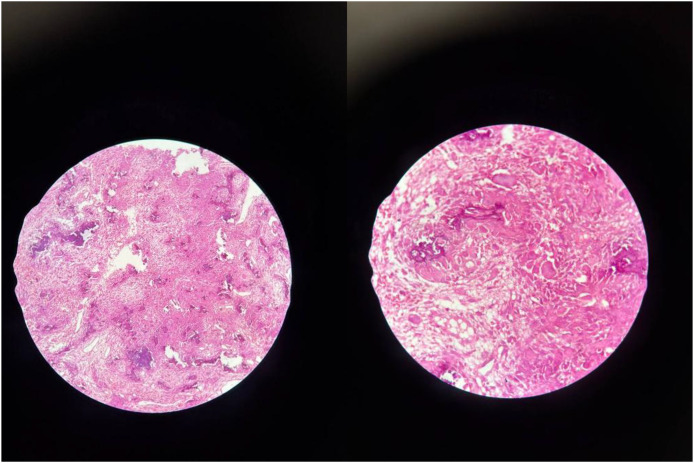
Histopathological section of the excised tissue demonstrating features of a ganglion cyst coexisting with osteoid osteoma in the head of the fifth metatarsal.

**Fig. 9:**
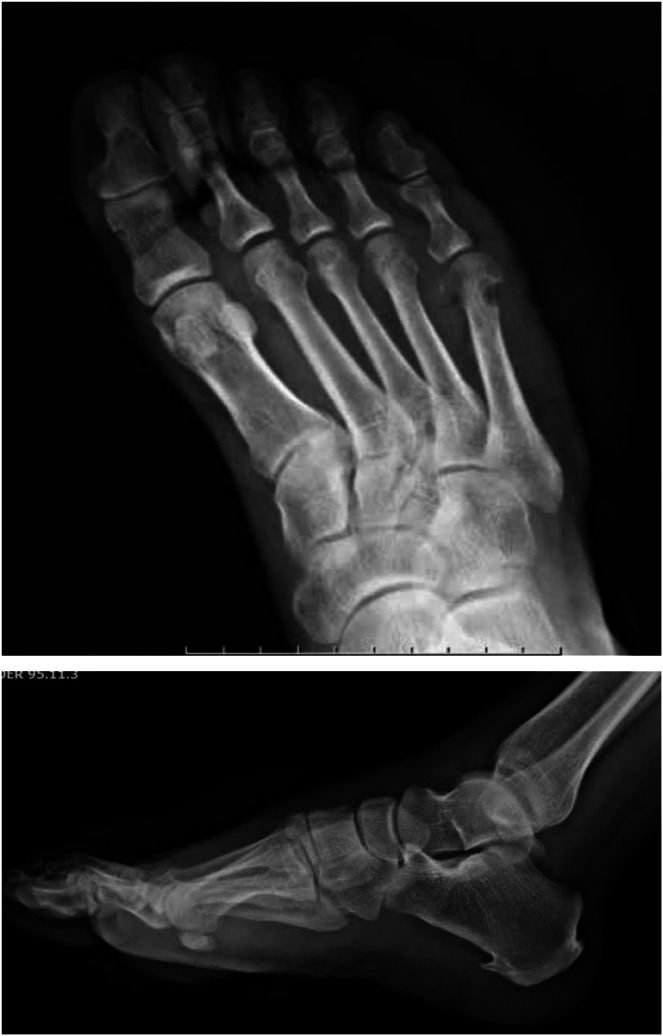
Postoperative dorsoplantar radiograph of the right foot showing complete removal of the lesion with restoration of cortical continuity.

## Discussion

Osteoid osteoma is a benign, painful, bone-producing tumor characterized by a nidus composed of osteoid tissue, osteoblasts, and variable amounts of fibrovascular stroma, typically surrounded by reactive osteosclerosis [7]. It accounts for 2%-3% of all primary bone tumors and 10%-14% of all benign bone tumors [8]. The tumor predominantly affects individuals aged 5-30 years, with a male-to-female ratio of approximately 2-3:1 [9]. Although almost any bone can be affected, the lower extremity is the most common site, with the femur and tibia accounting for approximately half of all cases. Within the foot, the talus is the most frequently involved bone (61%), followed by the calcaneus, phalanges, and metatarsals, and foot and ankle lesions collectively represent only 2%-11% of all osteoid osteomas [7,10]. The present case is notable for several reasons: the occurrence of osteoid osteoma in a 70-year-old man, its location in the medullary portion of the fifth metatarsal head, and its coexistence with a ganglion cyst—each of which is individually uncommon and, in combination, has not been previously reported.

Osteoid osteoma is characteristically a tumor of young adults. In the largest series of acral osteoid osteomas to date, Alruwaii et al. [8] reported 71 pathologically confirmed cases in the hands and feet with a median age of 23 years (range 7-64) [8]. Gurkan and Erdogan [9] reported a case series of 9 foot and ankle osteoid osteomas with a mean age of 21 years (range 6-30) [9]. Tomasian et al. [11] specifically studied osteoid osteoma in “older adults” (defined as ≥35 years) and demonstrated that percutaneous ablation remains effective in this population, but even in that cohort, the mean age was considerably younger than 70 years [11]. A vertebral osteoid osteoma has been reported in a 64-year-old man, and a patellar osteoid osteoma in a 51-year-old woman, but reports in the seventh decade of life or beyond remain exceedingly rare [12,13]. The present patient, diagnosed at age 70, therefore represents one of the oldest documented cases of osteoid osteoma and underscores that this diagnosis should not be excluded on the basis of age alone.

Osteoid osteoma of the fifth metatarsal is rare. Ataoglu et al. [10] reported a case of osteoid osteoma at the proximal diaphysis of the fifth metatarsal in a 29-year-old man who presented with a 2-year history of chronic lateral forefoot pain, nocturnal exacerbation, and relief with NSAIDs—features closely paralleling the present case [10]. Freschi and Dodson [14] described an osteoid osteoma of the second metatarsal treated with curettage, noting that forefoot osteoid osteoma is frequently subject to diagnostic delay [14]. In the present patient, the lesion was located in the medullary portion of the fifth metatarsal head, which is atypical. Medullary (cancellous) osteoid osteomas are less common than cortical lesions and tend to exhibit milder reactive sclerosis that may appear distant from the nidus, making radiographic detection more challenging [15,16]. This likely contributed to the prolonged symptom duration of several years in the present case, as the patient’s pain was partially controlled by NSAIDs and may have been misattributed to footwear-related pressure or degenerative causes. Such diagnostic delays are well-recognized in foot osteoid osteoma; Gurkan and Erdogan [9] emphasized that patients are often treated conservatively for prolonged periods, resulting in social, economic, and psychological consequences [9]. Similarly, He et al. [17] reported a case of talar osteoid osteoma that was misdiagnosed for 3 years before CT ultimately revealed the nidus [17].

In the present case, initial plain radiographs showed only a subtle cyst-like lesion on the lateral view, without obvious fracture or dislocation. CT subsequently demonstrated cortical thickening and a small lytic lesion, while MRI revealed a round, low-signal nidus on T1-weighted imaging. This stepwise imaging approach is consistent with current recommendations. The American College of Radiology Appropriateness Criteria designate CT without contrast as the optimal modality when osteoid osteoma is suspected, given its high sensitivity (96.4%) for nidus detection [18]. MRI, while valuable for evaluating soft tissue changes and bone marrow edema, has been reported to miss the diagnosis in approximately one-third of foot osteoid osteoma cases, largely because extensive perilesional edema can obscure the nidus [7,15]. Shukla et al. found that CT identified the nidus in 89% of foot osteoid osteomas, compared with only 67% for MRI [15]. In the present case, the complementary use of both modalities proved essential: CT delineated the bony lesion, while MRI characterized the nidus and the adjacent soft tissue ganglion cyst.

The patient underwent open surgical excision of the soft tissue mass with curettage of the nidus and cauterization of the inner walls, resulting in complete symptom resolution within 10 days. Open curettage remains a well-established treatment for foot and ankle osteoid osteoma, with reported success rates of 88%-100% [7,19]. Although percutaneous ablation techniques—particularly radiofrequency ablation—have become the preferred approach for osteoid osteoma in typical locations, open surgery remains a valuable option for lesions in atypical sites or near neurovascular structures [19,20]. In the present case, open surgery was selected because it allowed simultaneous excision of the coexisting ganglion cyst and direct curettage of the medullary nidus, which would not have been feasible with percutaneous ablation alone. Ataoglu et al. [10] similarly employed open surgical excision for their fifth metatarsal osteoid osteoma, achieving complete symptom relief [10]. The rapid postoperative resolution of symptoms in the present case is consistent with the literature, which consistently demonstrates that complete nidus removal is curative [20,21]

Ganglion cysts are benign soft tissue lesions filled with gelatinous fluid rich in hyaluronic acid and mucopolysaccharides, believed to arise from connective tissues of joint capsules and tendon sheaths through mechanisms including repetitive microtrauma, mucoid degeneration, or synovial herniation [18,22]. While ganglion cysts are common in the wrist and hand, those affecting the foot are relatively uncommon [22,23]. Wolf and Fallat [22] reported a case of an intermetatarsal ganglion cyst causing reactive osseous remodeling of adjacent metatarsals, demonstrating that soft tissue ganglia can interact with neighboring bone [22]. Intraosseous ganglia have been reported in the metatarsals, including a case in the second metatarsal treated with endoscopic curettage and one in the third metatarsal presenting with a pathological fracture [24,25]. However, the coexistence of a soft tissue ganglion cyst with an osteoid osteoma in the same anatomical region, as observed in the present case, has not been previously documented. Whether this association is coincidental or whether the chronic inflammatory milieu generated by the osteoid osteoma—characterized by elevated prostaglandin synthesis and local vasodilation—may have contributed to ganglion formation through mucoid degeneration of adjacent connective tissue remains speculative and warrants further investigation [26].

The literature on metatarsal osteoid osteoma consists predominantly of isolated case reports. Chapala et al. [27] recently published the largest cohort of metatarsal tumors (93 cases), in which osteoid osteoma was the most common diagnosis (10.8%), with 80% male predominance [27]. Alruwaii et al. [8] reported that osteoid osteomas of the hands and feet are frequently misdiagnosed, with clinical impressions often including neoplastic and inflammatory etiologies, and that cortical thickening and perilesional edema almost always exceeded the size of the nidus by at least twofold [8]. These observations are consistent with the present case, in which the initial differential diagnosis included degenerative and pressure-related etiologies before advanced imaging and histopathology confirmed the diagnosis. The present case extends the known clinical spectrum of osteoid osteoma by documenting its occurrence in an elderly patient, at an uncommon site, and in association with a ganglion cyst—a combination that, to our knowledge, has not been previously reported.

This report describes a single case, and the findings cannot be generalized. The pathogenetic relationship between the osteoid osteoma and the ganglion cyst remains unclear. Long-term follow-up data are not yet available to assess for recurrence, although recurrence rates following complete curettage of osteoid osteoma are generally low [8,19].

In conclusion, this case highlights the importance of maintaining a broad differential diagnosis for chronic foot pain, even in elderly patients in whom osteoid osteoma is not typically suspected. The combination of CT and MRI was essential for accurate diagnosis, and open surgical excision with curettage proved effective for simultaneous management of both lesions. The unprecedented coexistence of osteoid osteoma with a ganglion cyst in the fifth metatarsal adds to the growing body of literature on atypical presentations of this tumor and reinforces the value of documenting rare cases to guide future clinical practice.

## Ethical approval

The institutional ethics committee’s approval for this case report was obtained.

## Patient consent

Written informed consent was obtained from the patient for publication and any accompanying images. A copy of the written consent is available for review by the Editor-in-Chief of this journal on request.

## References

[1] Hawke F., Burns J. (2009). Understanding the nature and mechanism of foot pain. J Foot Ankle Res.

[2] Ruggieri P, Angelini A, Jorge FD, Maraldi M, Giannini S (2014). Review of foot tumors seen in a university tumor institute. J Foot Ankle Surg.

[3] Kitsoulis P., Mantellos G., Vlychou M. (2006). Osteoidosteoma. Acta Orthop Belg.

[4] Behzadi E., Mollaabbasi M. (2025). Presentation of osteoid osteoma in the fifth metatarsal: a case report and review of the literature. J Med Case Rep.

[5] Head L, Gencarelli JR, Allen M, Boyd KU (2015). Wrist ganglion treatment: systematic review and meta-analysis. J Hand Surg Am.

[6] Migonis A. (2019). A case report and literature review: intraneural ganglion cyst causing tarsal tunnel syndrome. J Foot Ankle Surg.

[7] Reda B. (2018). Cystic bone tumors of the foot and ankle. J Surg Oncol.

[8] Alruwaii F, Molligan JF, Ilaslan H, John I, Herradura AS, Alkashash A (2023). Osteoid osteomas of the hands and feet: a series of 71 cases. Virchows Arch.

[9] Gurkan V., Erdogan O. (2018). Foot and ankle osteoid osteomas. J Foot Ankle Surg.

[10] Ataoglu MB, Ali AK, Ozer M, Topcu HN, Cetinkaya M, Kulduk G (2017). Osteoid osteoma at the proximal diaphysis of the fifth metatarsal. J Am Podiatr Med Assoc.

[11] Tomasian A, Cazzato RL, Auloge P, Garnon J, Gangi A, Jennings JW (2020). Osteoid osteoma in older adults: clinical success rate of percutaneous image-guided thermal ablation. Clin Radiol.

[12] Scuotto A. (2002). Unusual manifestation of vertebral osteoid osteoma: case report. Eur Radiol.

[13] Vallianatos PG, Tilentzoglou AC, Seitaridis SV, Mahera HJ (2006). Osteoid osteoma of the patella: a case report. Knee Surg Sports Traumatol Arthrosc.

[14] Freschi S., Dodson N.B. (2007). Osteoid osteoma: an uncommon cause of foot pain. J Am Podiatr Med Assoc.

[15] Shukla S., Clarke A.W., Saifuddin A. (2010). Imaging features of foot osteoid osteoma. Skeletal Radiol.

[16] Napora J., Wałejko S., Mazurek T. (2023). Osteoid osteoma, a diagnostic problem: a series of atypical and mimicking presentations and review of the recent literature. J Clin Med.

[17] He H, Xu H, Lu H, Dang Y, Huang W, Zhang Q (2017). A misdiagnosed case of osteoid osteoma of the talus: a case report and literature review. BMC Musculoskelet Disord.

[18] Expert Panel on Musculoskeletal Imaging, Ahlawat S, Lenchik L, Baker JC, Allen H, Banks J, Florou V (2025). ACR appropriateness Criteria® suspected primary bone tumors: 2024 update. J Am Coll Radiol.

[19] Shu M., Ke J. (2022). The surgical management of osteoid osteoma: a systematic review. Front Oncol.

[20] Tepelenis K, Skandalakis GP, Papathanakos G, Kefala MA, Kitsouli A, Barbouti A (2021). Osteoid osteoma: an updated review of epidemiology, pathogenesis, clinical presentation, radiological features, and treatment option. In. Vivo.

[21] Atesok KI, Alman BA, Schemitsch EH, Peyser A, Mankin H (2011). Osteoid osteoma and osteoblastoma. J Am Acad Orthop Surg.

[22] Wolf J., Fallat L. (2020). Osseous remodeling secondary to a chronic intermetatarsal ganglion cyst: a case report. J Foot Ankle Surg.

[23] Rozbruch SR, Chang V, Bohne WH, Deland JT. (1998). Ganglion cysts of the lower extremity: an analysis of 54 cases and review of the literature. Orthopedics.

[24] Lui T.H. (2020). Endoscopic curettage and bone grafting of intraosseous ganglion of the second metatarsal. J Foot Ankle Surg.

[25] Wakabayashi I, Okada K, Hashimoto M, Sageshima M (1999). Intraosseous ganglion of the metatarsal bone. J Comput Assist Tomogr.

[26] Ghosh P.S., Mitra S., Moodley M. (2012). Pearls & oy-sters: osteoid osteoma of the scapula masquerading as neuralgic amyotrophy. Neurology.

[27] Chapala S, Gibson J, Kiyawat V, Nischal N, Chinniah P, Botchu R (2026). Metatarsal tumors: a comprehensive statistical overview and clinical analysis of the largest reported cohort. Skeletal Radiol.

